# Comparative Effects of Live and Heat-Killed *Lactobacillus* Formulations on DNCB-Induced Dermatitis-like Skin Inflammation in Mice

**DOI:** 10.3390/nu18152419

**Published:** 2026-07-24

**Authors:** Yu-Syuan Lin, Zhen-Shu Liu, Yu-Jie Yang, Po-Wen Chen

**Affiliations:** 1 Department of Veterinary Medicine, College of Veterinary Medicine, National Chung Hsing University, No.145 Xingda Rd., South Dist., Taichung 40227, Taiwan; positiveshower@gmail.com (Y.-S.L.); fast0983772349@gmail.com (Y.-J.Y.); 2 Department of Safety, Health and Environmental Engineering, Ming Chi University of Technology, New Taipei City 24301, Taiwan; zsliu@mail.mcut.edu.tw; 3 Center for Environmental Sustainability and Human Health, Ming Chi University of Technology, New Taipei City 24301, Taiwan; 4 Chronic Diseases and Health Promotion Research Center, Chang Gung University of Science and Technology, Chiayi 61363, Taiwan

**Keywords:** atopic dermatitis-like inflammation, DNCB, *Lactobacillus gasseri*, heat-killed probiotics, paraprobiotics, mast cells, skin inflammation, generalized estimating equations

## Abstract

Background/Objectives: Atopic dermatitis (AD) is a chronic inflammatory skin disease involving barrier dysfunction, pruritus, and immune dysregulation, and microbial-based interventions are being explored as supportive approaches. This study first conducted an exploratory pilot model-development experiment to characterize DNCB-induced dermatitis-like inflammation and subsequently compared oral live *Lactobacillus*, heat-killed *Lactobacillus* (paraprobiotics), and a combined live/heat-killed formulation after disease induction. Methods: Male BALB/c mice received repeated topical DNCB exposure followed by 21 days of oral intervention. Outcomes included gross lesion scores, cumulative scratching time, ear and spleen indices, histopathology, qualitative mast-cell staining in the therapeutic experiment, and serum IgE, IL-4, and IFN-γ. Longitudinal gross scores were reanalyzed using generalized estimating equations with group, time, and group-by-time interaction. Results: After Holm correction, the mixed formulation showed a lower left-ear erythema score than placebo at Week 2, whereas no other intervention-versus-placebo gross-score comparison remained significant. Paraprobiotic and mixed formulations reduced spleen weight, but histopathological and serum biomarker responses were variable. Conclusions: These findings indicate partial, endpoint-dependent effects of live and heat-killed *Lactobacillus* formulations in DNCB-induced dermatitis-like inflammation.

## Simple Summary

Atopic dermatitis is a long-lasting inflammatory skin disease that causes itching, skin injury, and repeated flare-ups. Probiotics may help regulate inflammatory responses, but it is still unclear whether live bacteria, heat-killed bacteria, hereafter referred to as paraprobiotics, or a combination of both have different effects after skin inflammation has already developed. In this study, an exploratory pilot experiment was first used to characterize DNCB-induced dermatitis-like inflammation, followed by a therapeutic experiment comparing oral live probiotics, paraprobiotics, and a mixed formulation. The intervention effects were endpoint-dependent. The mixed formulation produced a transient reduction in left-ear erythema at Week 2, whereas paraprobiotic and mixed formulations reduced spleen weight relative to the Placebo + DNCB group. Most other gross lesion, histopathological, and serum biomarker comparisons did not clearly distinguish the intervention groups from the placebo group. These findings suggest that live probiotic and paraprobiotic preparations warrant further investigation as supportive approaches for inflammatory skin conditions.

## 1. Introduction

Atopic dermatitis (AD) is a chronic and relapsing inflammatory skin disease characterized by epidermal barrier dysfunction, intense pruritus, and immune dysregulation. It is commonly associated with elevated IgE levels and type 2 immune responses, particularly Th2-associated cytokines such as IL-4 and IL-13, which contribute to barrier impairment and allergic inflammation [1,2]. In chronic or recurrent disease stages, additional immune pathways, including Th1 and Th17/Th22 responses, may further contribute to persistent inflammation, epidermal hyperplasia, and tissue remodeling [3,4].

AD substantially impairs quality of life through persistent itching, sleep disturbance, recurrent skin lesions, and psychological burden. Current therapeutic strategies include topical corticosteroids, immunosuppressants, and emerging biologics targeting type 2 inflammation. Although these treatments are effective in many patients, their long-term use may be limited by adverse effects, cost, accessibility, or incomplete disease control, particularly in moderate-to-severe or recurrent cases [5,6]. Therefore, adjunctive or alternative approaches that can modulate inflammatory responses and support skin recovery remain of interest.

In recent years, microbiota-targeted interventions, including probiotics, have been investigated for their immunomodulatory potential in allergic and inflammatory diseases, including AD. However, clinical and preclinical outcomes remain inconsistent, largely due to strain-specific effects, formulation variability, differences in disease severity, and limited efficacy in established disease settings [7,8,9,10,11]. In addition, the biological activity of live probiotics may be influenced by viability, storage stability, gastrointestinal survival, and host-dependent colonization conditions. In contrast, heat-killed probiotic preparations are less affected by viability-related limitations and may offer improved stability and safety. More recently, heat-killed probiotics, hereafter referred to as paraprobiotics, have emerged as alternative microbial-based interventions. Evidence suggests that inactivated bacteria may still exert immunomodulatory effects through microbial structural components and host pattern recognition receptors, without requiring colonization or active metabolic activity [12,13].

Importantly, many preclinical studies of probiotic-based interventions in AD have focused on preventive administration or relatively mild inflammatory models. In comparison, the effects of live probiotic and paraprobiotic formulations under established inflammatory conditions remain insufficiently characterized. This distinction is important because therapeutic intervention after disease establishment may better reflect clinical situations in which treatment begins after skin inflammation and pruritus have already developed. In addition, chemically induced dermatitis models, such as DNCB-induced AD-like models, may vary in lesion severity and tissue damage depending on the induction schedule and local exposure conditions. A preliminary model-development experiment can therefore help characterize lesion patterns and inform a subsequent therapeutic protocol.

Accordingly, this study aimed to (i) conduct an exploratory pilot experiment to characterize dermatitis-like inflammation produced by different DNCB exposure schedules and use those observations to inform the subsequent protocol and (ii) evaluate and compare the effects of oral live probiotics, paraprobiotics, and a combined live/paraprobiotic formulation after disease establishment. By comparing these formulations under established inflammatory conditions, this study sought to clarify whether paraprobiotic preparations retain biologically relevant activity and whether combined live and heat-killed formulations produce different responses across behavioral, gross lesion, and histopathological endpoints.

## 2. Materials and Methods

### 2.1. Probiotic Strains and Preparation

The probiotic formulation used in this study consisted of three human breast milk-derived *Lactobacillus gasseri* strains designated HM1-LGA, HM1-LGC, and HM1-LGD, which were originally selected and characterized for potential application in urinary tract infection prevention and are associated with Taiwan Invention Patent No. I928791. The three strains were combined using a fixed proprietary formulation ratio. Because the exact ratio forms part of ongoing intellectual-property development for a separate formulation-related application, it is not disclosed; consequently, exact independent reproduction of the formulation ratio is currently limited. For bacterial preparation, *Lactobacillus* strains were cultured in de Man, Rogosa and Sharpe (MRS) broth (BD Biosciences, San Jose, CA, USA) under anaerobic conditions at 37 °C for 24 h. Bacterial cells were harvested by centrifugation at 12,000× *g* for 10 min, washed three times with phosphate-buffered saline (PBS), and resuspended in sterile PBS for oral administration. The bacterial concentration was adjusted to approximately 1 × 10^9^ CFU/mL before administration.

A single standardized bacterial suspension was divided into two matched aliquots. One aliquot was retained as the live preparation, whereas the other was heat-inactivated at 121 °C and 1.5 atm for 15 min. Thus, the live and heat-killed preparations originated from the same culture batch and underwent identical culture, harvesting, washing, and resuspension procedures before heat treatment. Viable counts of the live preparation were reconfirmed before administration, and complete heat killing was verified by the absence of colonies on MRS agar. Dose matching was based on pre-inactivation CFU equivalents rather than dry weight or protein content. The live group received approximately 2 × 10^8^ viable CFU/mouse/day, and the paraprobiotic group received approximately 2 × 10^8^ pre-inactivation CFU equivalents/mouse/day. The mixed formulation was prepared by combining equal volumes of the matched live and heat-killed preparations and provided approximately 1 × 10^8^ viable CFU plus 1 × 10^8^ heat-killed CFU equivalents/mouse/day, maintaining the same total bacterial-equivalent dose. All formulations were administered once daily by oral gavage at 0.2 mL/mouse/day.

### 2.2. Animals and Ethics Statement

All animal procedures were approved by the Institutional Animal Care and Use Committee of National Chung Hsing University (IACUC No. 112-123R). Six-week-old male BALB/c mice were obtained from the National Laboratory Animal Center (Taiwan). Male mice were used to maintain consistency with previously reported DNCB protocols and the pilot experiment and to reduce variability associated with sex-related hormonal cycling. Mice were housed at 4–5 animals per cage under controlled environmental conditions (22–26 °C, 50–70% relative humidity, and a 12 h light/dark cycle) with wood-chip bedding, wooden-block enrichment, LabDiet 5001 rodent diet, and water ad libitum. Animals were acclimatized for 2 weeks. Simple random allocation to experimental groups was performed after acclimatization. Treatment administration was not blinded; however, coded clinical images and histopathological sections were evaluated by assessors blinded to group allocation. No formal prospective power calculation was performed; group sizes were selected based on comparable published DNCB studies, the exploratory design, and animal-welfare considerations. Animals were monitored according to institutional pain and distress guidelines. Predefined humane endpoints included complete loss of appetite for 24 h or food intake below 50% of the normal level for three consecutive days. No animals died or reached a humane endpoint before the scheduled endpoint. At study termination, mice were deeply anesthetized with intraperitoneal Zoletil-50 (Virbac, Milperra, Australia), followed by terminal cardiac blood collection and exsanguination.

### 2.3. DNCB-Induced Dermatitis-like Skin Inflammation Model

2,4-Dinitrochlorobenzene (DNCB; Sigma-Aldrich, St. Louis, MO, USA) was dissolved in acetone/olive oil (4:1, *v*/*v*) and used to induce AD-like skin inflammation.

#### 2.3.1. Exploratory Pilot Model-Development Experiment

An exploratory pilot experiment was conducted to characterize lesion development under two DNCB schedules. Mice were randomly divided into three groups (*n* = 6/group): healthy control, continuous DNCB induction, and intermittent DNCB induction. The continuous group received 1% DNCB daily for four consecutive days on the dorsal skin (100 μL under occlusive dressing) and right ear (20 μL without occlusion) and was euthanized on Day 8. The intermittent group received 1% DNCB on Days 1 and 4 and 0.2% DNCB on Days 7 and 10 at the same sites and volumes and was euthanized on Day 11. Healthy controls received vehicle. Because the groups were evaluated at different terminal time points, this pilot experiment was used to characterize lesion patterns and practical model performance rather than to establish formal superiority of one schedule.

#### 2.3.2. Therapeutic Experiment

Based on observations from the exploratory pilot experiment, the induction procedure was empirically refined for the therapeutic experiment by reducing the initial induction from four to three consecutive days to avoid excessively severe tissue injury and retain an observable range for intervention-associated changes. Mice were randomly assigned to five groups: healthy control (*n* = 7), Placebo + DNCB (*n* = 10), Probiotic + DNCB (*n* = 10), Paraprobiotic + DNCB (*n* = 10), and Mix + DNCB (*n* = 10). Before DNCB induction, dorsal hair was removed using depilatory cream in all groups, including the healthy control group. On Days 1–3, 1% DNCB was applied once daily to the dorsal skin (100 μL under occlusive dressing) and dorsal surface of the left ear (20 μL without occlusion); healthy controls received vehicle. After induction, mice received the assigned oral formulation for 21 days. On Day 25, the same left ear was rechallenged with 100 μL of 0.5% DNCB. No occlusive dressing was applied to the ear, and the solution was left in place without washing until sacrifice approximately 24 h later. The contralateral right ear received no local DNCB and served as a non-challenged comparison. Healthy controls received corresponding vehicle applications. All mice were euthanized on Day 26. The therapeutic protocol was informed by, but was not identical to or independently validated after, the pilot protocol.

### 2.4. Clinical Scoring and Scratching Behavior

Gross skin severity was evaluated from coded photographs by two independent assessors blinded to treatment allocation, one of whom had prior training in dermatological scoring. The evaluated parameters included erythema, dryness, edema, and erosion/excoriation, each scored on a 0–3 scale. The total clinical score was calculated as the sum of individual parameter scores. Scratching behavior was recorded for 10 min before sacrifice and quantified as cumulative scratching time from coded video recordings by blinded assessors.

### 2.5. Body Weight and Organ Index

Body weight was recorded daily in the exploratory pilot experiment and weekly in the therapeutic experiment. Left and right ears were collected and analyzed separately, and spleens were collected at sacrifice. Organ indices were calculated as tissue weight divided by body weight and expressed as mg/g. In the therapeutic experiment, left-ear outcomes represented the locally challenged site, whereas the right ear represented the non-challenged contralateral site.

### 2.6. Histological Analysis

Histopathological severity was evaluated by a blinded veterinary pathologist, who independently scored all tissue sections using a predefined 5-grade scoring system based on lesion extent and severity. The evaluated parameters included hyperkeratosis, epidermal hyperplasia, serocellular pustule formation, inflammatory cell infiltration, ulceration, and necrosis. Representative histological images were used only to illustrate individual histopathological features; group-level interpretation was based on the quantitative scores, incidence data, and statistical comparisons presented in Table 1, Table 2 and Table 3. One dorsal-skin section from the paraprobiotic group was unsuitable for reliable pathological evaluation and was excluded, resulting in *n* = 9 for that tissue analysis. Ear thickness was additionally measured using a digital micrometer.

### 2.7. Mast-Cell Staining

Mast cells were detected using 0.1% toluidine blue staining. In the exploratory pilot experiment, mast cells were quantified in five randomly selected fields per tissue section at 400× magnification and expressed as the mean number of mast cells per field. In the therapeutic experiment, toluidine blue-stained sections were examined qualitatively, and representative images were used only to illustrate mast-cell distribution; no formal group-level quantitative comparison was performed.

### 2.8. ELISA

To evaluate systemic immune responses, serum samples were collected at sacrifice on Day 26 for enzyme-linked immunosorbent assay (ELISA) analysis. Serum concentrations of total IgE, interleukin-4 (IL-4), and interferon-gamma (IFN-γ) were measured using commercially available ELISA kits from Invitrogen (Thermo Fisher Scientific, Waltham, MA, USA). Total IgE was measured using the Mouse IgE ELISA Kit (Cat. No. EMIGHEX10; detection range, 0.137–100 ng/mL; analytical sensitivity, 0.14 ng/mL). IL-4 was measured using the Mouse IL-4 ELISA Kit (Cat. No. BMS613; detection range, 3.9–250 pg/mL; analytical sensitivity, 2.0 pg/mL). IFN-γ was measured using the Mouse IFN-γ ELISA Kit (Cat. No. KMC4021; detection range, 4.7–300 pg/mL; analytical sensitivity, <2 pg/mL). Because analyte concentrations varied among serum samples, preliminary assays were performed and dilution factors were adjusted, when necessary, to ensure that absorbance values fell within the calibration range of the corresponding kit; therefore, a single fixed serum dilution factor was not uniformly applied to all analytes or samples. All assays were performed according to the manufacturer’s instructions. Individual mice were considered the biological replicates. Optical density was measured at 450 nm, and concentrations were calculated from standard curves generated using the standards supplied with each kit. Values below the assay-specific lower detection limit were classified as below detection and were not assigned numerical concentrations or included in quantitative group comparisons.

### 2.9. Statistical Analysis

Statistical analyses were performed using IBM SPSS Statistics for Windows, version 20.0 (IBM Corp., Armonk, NY, USA). Data are presented as mean ± standard deviation (SD). Terminal endpoints measured once at sacrifice, including cumulative scratching time, organ indices, serum biomarkers, and histopathological scores, were analyzed by one-way analysis of variance (ANOVA) followed by LSD post hoc testing. Longitudinal left-ear erythema, right-ear erythema, and dorsal gross lesion scores from Weeks 1–4 were reanalyzed using generalized estimating equations (GEE) with an exchangeable working-correlation structure and robust standard errors. The models included group, time, and group-by-time interaction, with mouse identity specified as the repeated subject. Post hoc comparisons between each intervention group and the Placebo + DNCB group at each week were adjusted using the Holm procedure. Additional Kruskal–Wallis analyses were used as sensitivity analyses for ordinal or semi-quantitative terminal scores. A two-sided *p*-value < 0.05 was considered statistically significant.

## 3. Results

### 3.1. Exploratory Pilot Characterization of DNCB-Induced Dermatitis-like Inflammation

**Figure 1:**
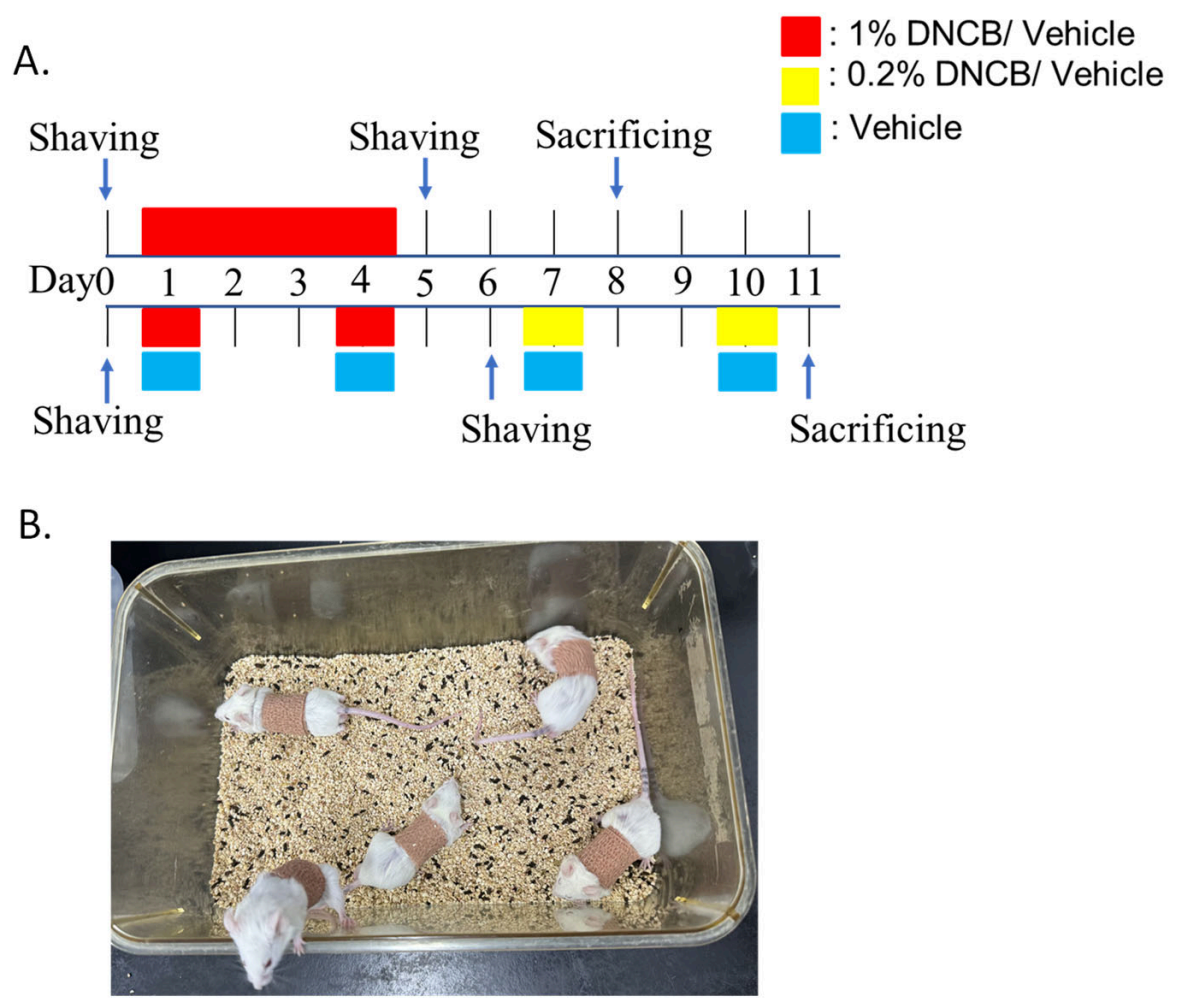
Experimental design and establishment of the DNCB-induced dermatitis model. (**A**) Experimental schedule and grouping of mice used for establishment of the DNCB-induced dermatitis model. (**B**) Representative photograph showing occlusive bandaging of the dorsal skin following topical DNCB application.

**Figure 2:**
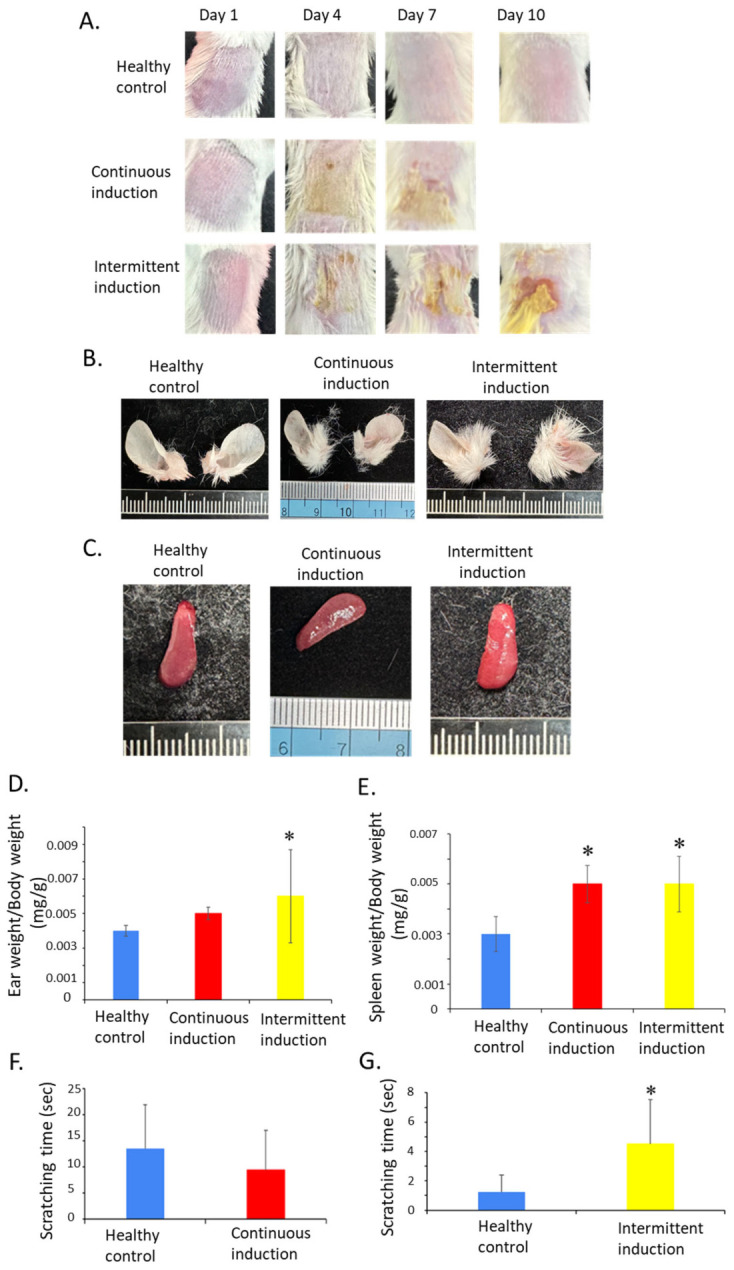
Clinical characteristics of the DNCB-induced dermatitis model. (**A**) Representative dorsal skin appearances of mice in the continuous induction and intermittent induction groups at different experimental time points. (**B**) Representative images of bilateral ears from mice in the continuous induction group (left), intermittent induction group (middle), and healthy control group (right). DNCB was applied only to the right ear to evaluate localized inflammatory responses. (**C**) Representative gross morphology of spleens collected from the three experimental groups. (**D**) Comparison of normalized right-ear weights among the experimental groups. (**E**) Comparison of spleen weights normalized to body weight among the experimental groups. (**F**) Scratching behavior on day 7 in the healthy control and continuous induction groups. (**G**) Scratching behavior on day 10 in the healthy control and intermittent induction groups. Data are presented as mean ± SD (*n* = 6). * *p* < 0.05 versus the healthy control group.

The exploratory pilot experiment compared continuous and intermittent DNCB induction protocols to characterize lesion development and practical model performance, and an occlusive elastic bandage was applied to improve local retention and exposure consistency (Figure 1A,B). Both induction protocols produced dermatitis-like lesions, including erythema, scaling, excoriation, crust formation, and visible tissue damage (Figure 2A). Ear swelling was increased in DNCB-treated mice, particularly in the right ear, and splenomegaly was also observed in both induction groups compared with healthy controls (Figure 2B,C). Because the continuous and intermittent groups were euthanized on different days, these observations were not used to claim formal superiority, greater reproducibility, or greater severity of either schedule.

Quantitative analysis showed that normalized right-ear weight was increased in DNCB-treated mice, and spleen weight relative to body weight was also elevated (Figure 2D,E). Scratching time was increased under the evaluated induction conditions (Figure 2F,G). Histopathological examination demonstrated epidermal hyperplasia, hyperkeratosis, serocellular pustule formation, necrosis, and inflammatory cell infiltration in the dermal and subcutaneous layers (Figures S1A and S2A). The pilot observations informed empirical refinement of the subsequent therapeutic protocol; they were not interpreted as independent validation of a finalized induction schedule.

**Figure 3:**
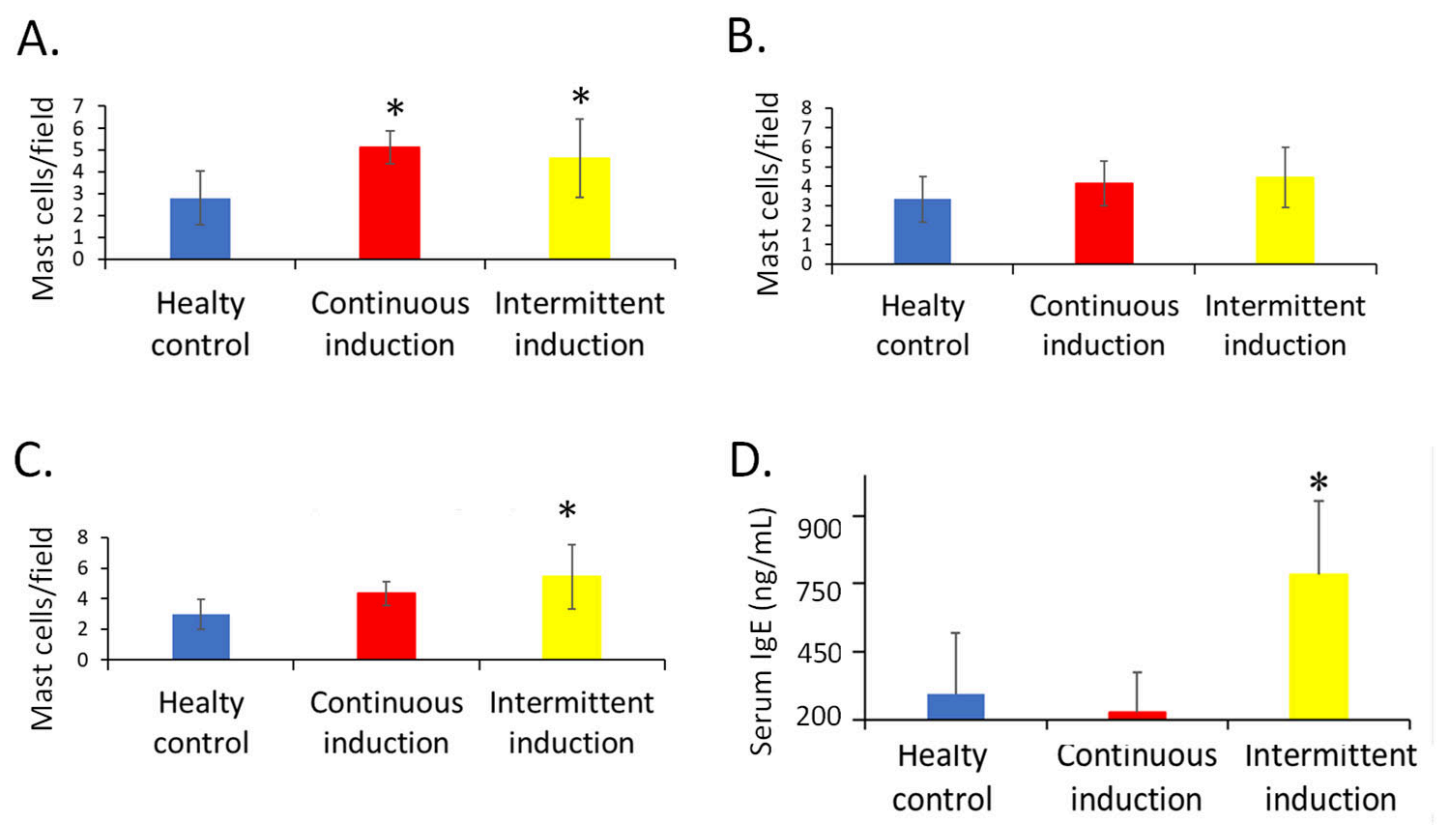
Mast-cell infiltration and serum IgE levels following establishment of the dermatitis model. Toluidine blue staining was performed to evaluate mast-cell infiltration in dorsal skin (**A**), left-ear (**B**), and right-ear (**C**) tissues from each experimental group. Mast-cell numbers were quantified microscopically. (**D**) Serum IgE concentrations among the experimental groups. Data are presented as mean ± SD (*n* = 6). * *p* < 0.05 versus the healthy control group.

Toluidine blue staining and formal mast-cell counting in the exploratory pilot experiment showed increased mast-cell numbers in selected DNCB-treated tissues (Figures S1B and S2B and Figure 3A–C). Serum IgE was also elevated in the intermittent-induction group (Figure 3D). These pilot findings were used to characterize the inflammatory phenotype and inform the subsequent therapeutic design.

### 3.2. Effects of Live Probiotics, Paraprobiotics, and the Combined Formulation on DNCB-Induced Dermatitis-like Symptoms

**Figure 4:**
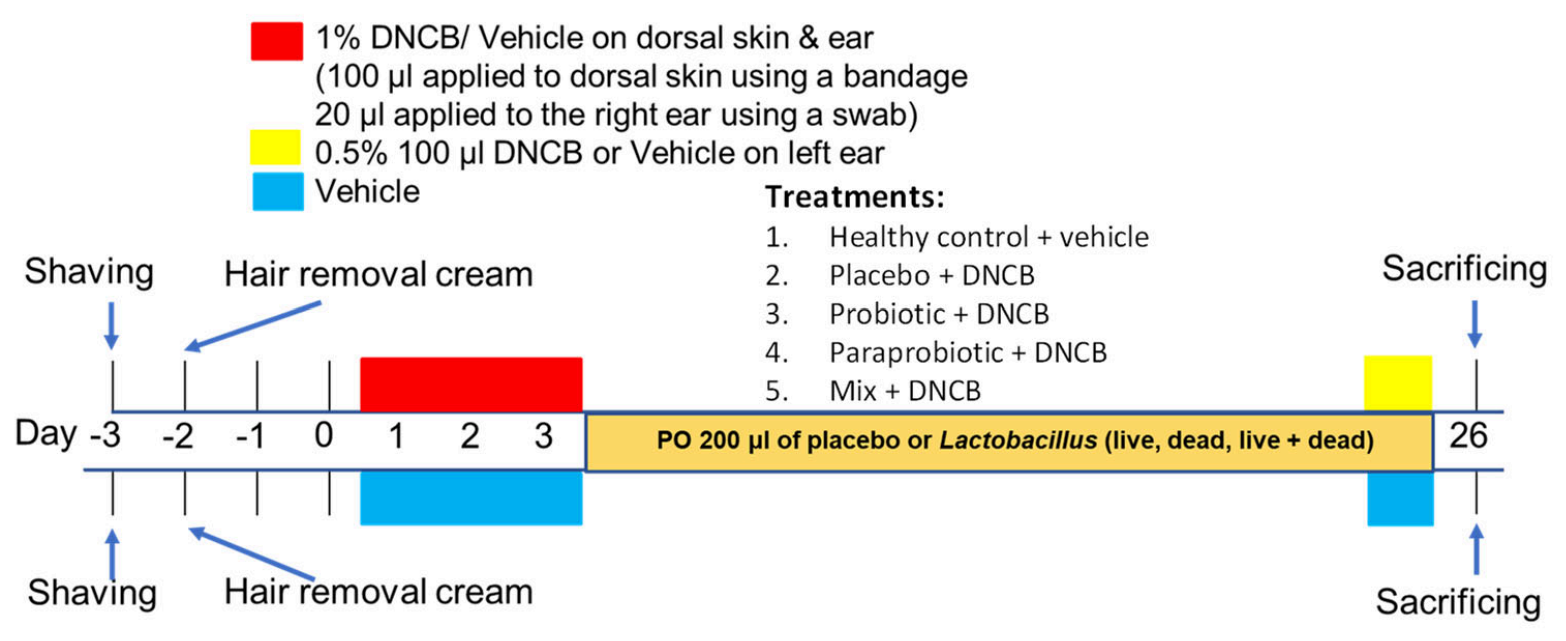
Experimental design and timeline of the therapeutic experiment in the DNCB-induced dermatitis model. A total of 47 male BALB/c mice were randomly assigned into five groups: healthy control (*n* = 7), Placebo + DNCB (*n* = 10), Probiotic + DNCB (*n* = 10), Paraprobiotic + DNCB (*n* = 10), and Mix + DNCB (*n* = 10). After acclimation, dorsal hair was removed prior to DNCB induction, and mice were sacrificed on day 26. Mice in the DNCB-treated groups received topical DNCB induction followed by a secondary ear challenge before sacrifice, whereas healthy control mice received vehicle treatment only. Repeated shaving during the experimental period was avoided to allow observation of hair regrowth among groups. The Probiotic + DNCB group received oral administration of live *Lactobacillus*, the Paraprobiotic + DNCB group received heat-killed *Lactobacillus*, and the Mix + DNCB group received combined administration of live and heat-killed *Lactobacillus*. Control groups received sterile PBS.

**Figure 5:**
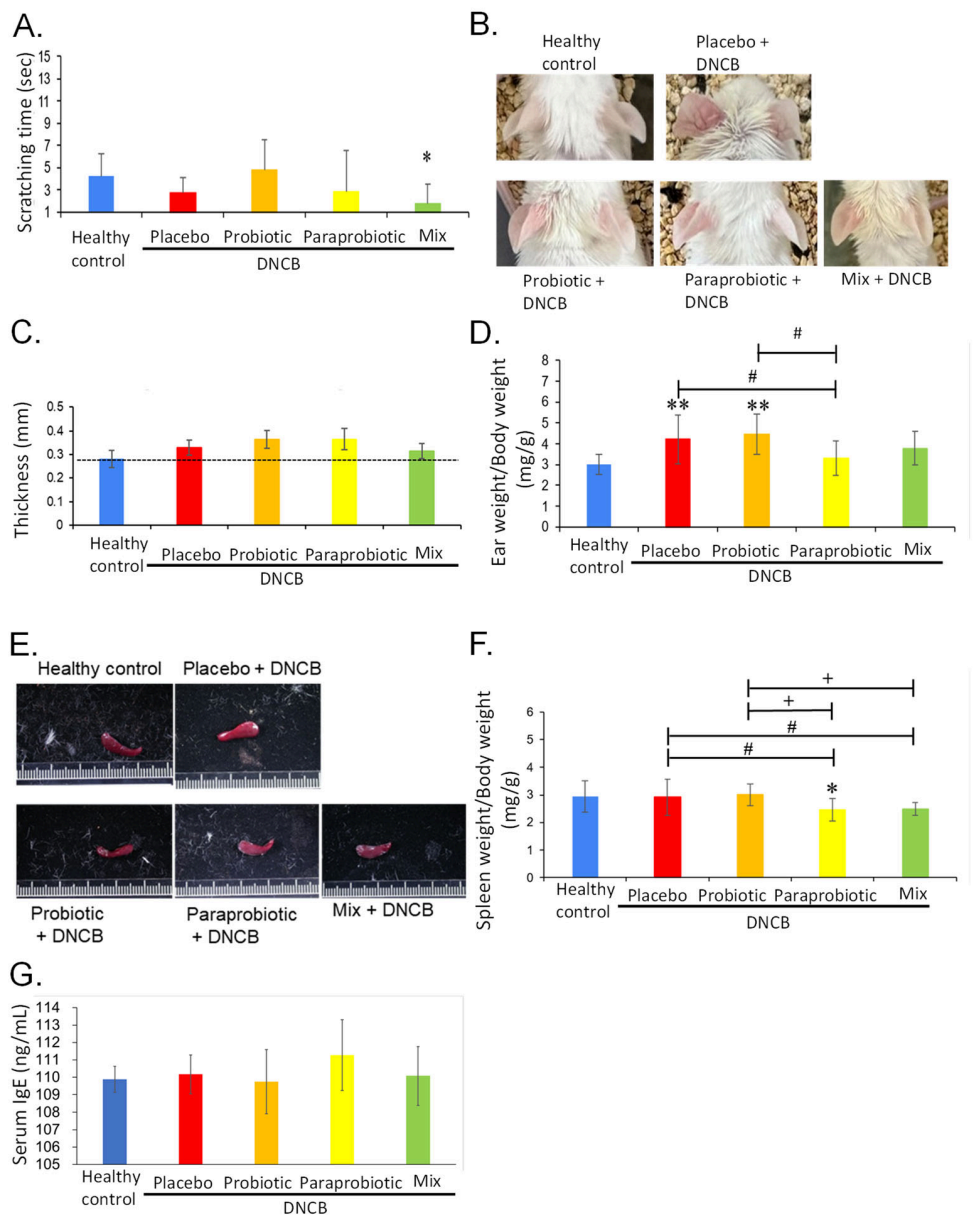
Effects of different probiotic formulations on behavioral and inflammatory phenotypes in DNCB-induced dermatitis-like mice. (**A**) Cumulative scratching time recorded during the 10 min observation period prior to sacrifice. (**B**) Representative images of left and right ears from each experimental group. (**C**) Ear thickness of the left ear following secondary DNCB challenge. (**D**) Left-ear weight normalized to body weight. (**E**) Representative gross appearance of spleens from each group. (**F**) Spleen weight normalized to body weight. (**G**) Serum IgE concentration. Data are presented as mean ± SD. # *p* < 0.05 versus Placebo + DNCB; + *p* < 0.05 versus Probiotic + DNCB. * *p* < 0.05 and ** *p* < 0.01 versus the healthy control group. The dashed line indicates the reference value for the corresponding measurement.

Mice were orally administered live probiotics, paraprobiotics, a combined formulation, or placebo after DNCB induction (Figure 4). As shown in Figure 5A, cumulative scratching time was assessed for 10 min before sacrifice. The Mix + DNCB group showed the lowest absolute scratching time among the DNCB-induced groups and was significantly lower than the Probiotic + DNCB group (*p* < 0.05). However, the difference between the Mix + DNCB and Placebo + DNCB groups did not reach statistical significance; therefore, this result does not establish an intervention-associated reduction relative to the placebo condition.

Left and right ears were analyzed separately. Because the right ear was not subjected to local DNCB challenge in the therapeutic experiment, subsequent analysis focused on the left ear, which received both primary induction and secondary challenge (Figure 5B–D). The Paraprobiotic + DNCB group showed significantly reduced left-ear weight compared with the Placebo + DNCB group (# *p* < 0.05), whereas the Mix + DNCB group showed a decreasing trend.

Spleen morphology and weight were evaluated as indicators of inflammation-associated systemic response (Figure 5E,F). The Healthy control group showed normal spleen morphology, whereas the Placebo + DNCB group exhibited apparent splenomegaly. In contrast, all probiotic-based treatment groups showed reduced spleen enlargement, with significantly lower spleen weights observed in the Paraprobiotic + DNCB and Mix + DNCB groups compared with the Placebo + DNCB group.

Serum biomarker analysis showed elevated IgE levels in DNCB-induced groups compared with the Healthy control group; however, high inter-individual variability prevented statistical significance among the treatment groups (Figure 5G). Serum IL-4 and IFN-γ levels were below the detection limit in all groups.

### 3.3. Longitudinal Gross Lesion and Ear Erythema Scores in DNCB-Induced Dermatitis-like Mice

Dorsal skin lesions and ear erythema were evaluated longitudinally during the therapeutic experiment. Week 1 corresponded to the initial induction period with 1% DNCB before intervention, Week 2 marked the initiation of placebo or probiotic-based treatment, and Week 4 represented the day after the secondary DNCB challenge and one day before sacrifice.

Representative gross lesion images showed that the Healthy control group remained lesion-free, whereas all DNCB-induced groups developed dermatitis-like changes after induction (Figure S3). Dorsal gross lesion scores declined over time in all DNCB-treated groups, and some intervention groups showed numerically lower scores than the Placebo + DNCB group at selected weeks. Generalized estimating equation analysis identified a significant group-by-time interaction (*p* < 0.001); however, after Holm correction, no intervention-versus-placebo comparison remained statistically significant at any evaluated week. These findings therefore indicate an overall improvement trend over time, but do not establish a statistically significant treatment-specific benefit relative to the Placebo + DNCB group.

**Figure 6:**
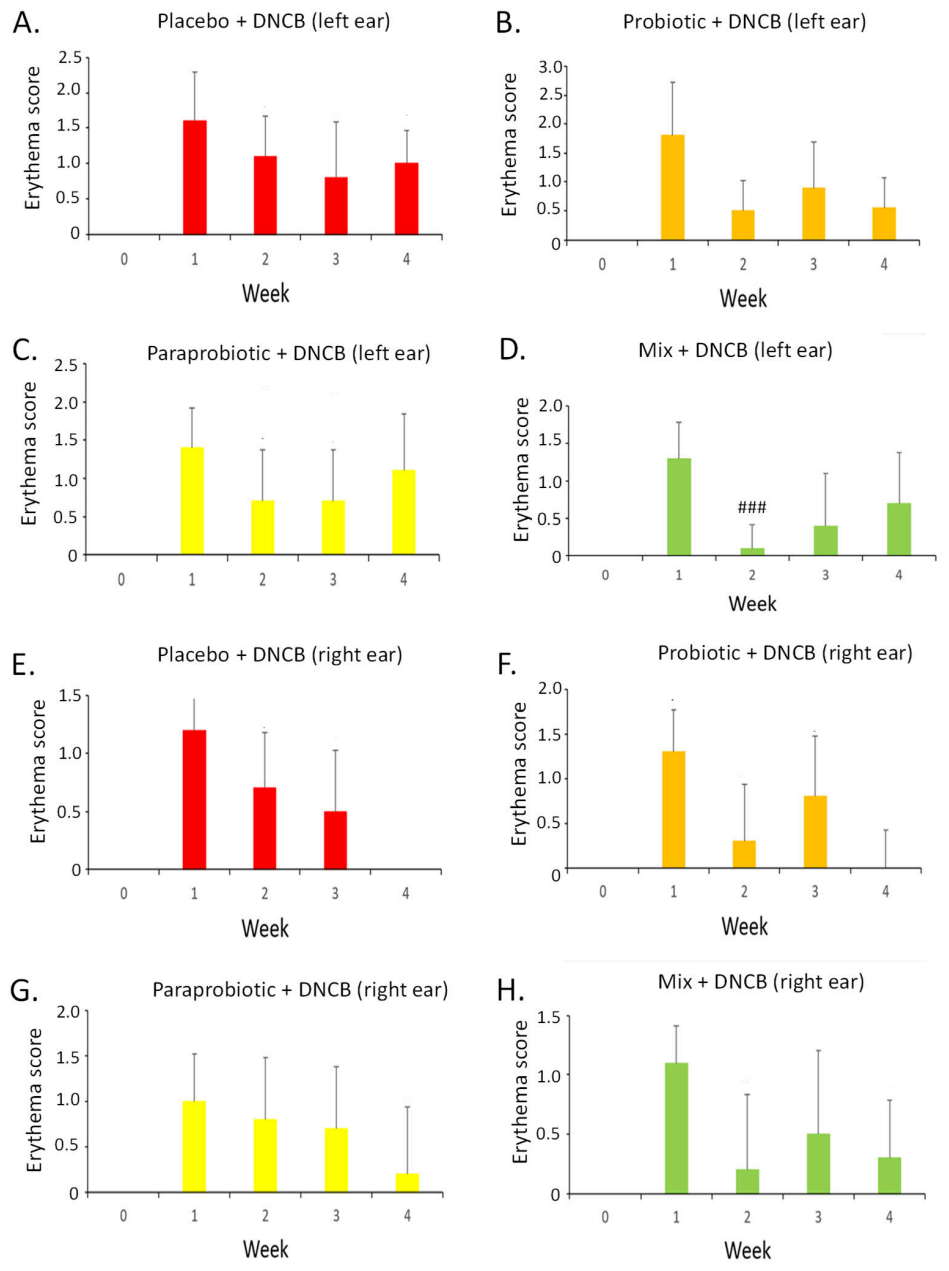
Gross erythema scores of the left- and right-ear skin in the probiotic-treated DNCB-induced dermatitis-like mouse model. (**A**–**D**) Erythema scores of the left ears in each experimental group; (**E**–**H**) erythema scores of the right ears. Week 1 corresponds to the initial induction period with 1% DNCB before probiotic intervention. Week 2 marks the initiation of placebo or probiotic administration. Week 4 represents the day after the secondary DNCB challenge and one day before sacrifice. Data are presented as mean ± standard deviation (SD). Longitudinal scores from Weeks 1–4 were analyzed using generalized estimating equations with group, time, and group-by-time interaction. Post hoc comparisons between each intervention group and the Placebo + DNCB group at each week were adjusted using the Holm procedure. Only significant Holm-adjusted between-group comparisons are shown. ### *p* < 0.001 versus Placebo + DNCB at the same week.

For ear erythema, generalized estimating equation analysis identified a significant group-by-time interaction for the left ear. After Holm correction, only the Mix + DNCB group at Week 2 showed a significantly lower left-ear erythema score than the Placebo + DNCB group (adjusted *p* = 3.48 × 10^−6^; Figure 6A–D). No other adjusted intervention-versus-placebo comparison was significant, including comparisons after the secondary challenge.

A significant group-by-time interaction was also detected for right-ear erythema; however, no intervention-versus-placebo comparison remained significant after Holm correction (Figure 6E–H). The longitudinal findings therefore support a transient difference in left-ear erythema for the mixed formulation at Week 2 rather than sustained differences across the study period.

### 3.4. Histopathological and Mast-Cell Evaluation

Histopathological analysis of ear and dorsal skin tissues revealed typical dermatitis-like changes in DNCB-treated mice, including epidermal hyperkeratosis, hyperplasia, pustule formation, necrosis, and inflammatory cell infiltration (Table 1, Table 2 and Table 3; Figures S4 and S5). Group-level interpretation was based on quantitative lesion scores, incidence data, and statistical comparisons in Table 1, Table 2 and Table 3; representative images were used only as illustrative examples.

In dorsal skin, the quantitative findings are presented in Table 3, whereas Figure S4 provides representative histological examples. Epidermal hyperplasia was observed in the DNCB-treated groups. Dorsal dermal and subcutaneous inflammation was significantly higher in the Paraprobiotic + DNCB group than in the Placebo + DNCB group, whereas the remaining intervention-versus-placebo comparisons were not statistically significant.

In ear tissues, quantitative lesion scores and incidence data are presented in Table 1 and Table 2, and Figure S5 provides representative examples. The unchallenged right ear showed minimal changes across groups. The challenged left ear showed DNCB-associated hyperkeratosis, epidermal hyperplasia, inflammation, and ulcerative/necrotizing lesions. Numerical differences among treatment groups were not interpreted as statistically supported treatment effects unless indicated by the corresponding analyses.

Detailed histopathological scoring showed minimal changes in the unchallenged right ear (Table 1). In the challenged left ear, DNCB-associated lesions were observed across DNCB-treated groups (Table 2). Although numerical differences in incidence and mean scores were present, most intervention-versus-placebo comparisons were not statistically significant and were therefore interpreted cautiously.

Histopathological evaluation of dorsal skin (Table 3) showed that DNCB treatment induced epidermal hyperplasia and inflammatory changes. One dorsal-skin section from the Paraprobiotic + DNCB group was unsuitable for reliable pathological evaluation, resulting in *n* = 9 for this tissue analysis.

Toluidine blue-stained dorsal-skin sections from the therapeutic experiment are shown in Figure S6 as representative qualitative observations of mast-cell distribution. No formal group-level mast-cell quantification was performed for this experiment; therefore, the images were not used to infer treatment-associated reductions in mast-cell infiltration.

Representative toluidine blue-stained ear sections are shown in Figure S7. These images were also evaluated qualitatively and were used only to illustrate mast-cell distribution in the challenged left ear and non-challenged right ear. No quantitative intergroup conclusions were drawn from these images.

Collectively, group-level histopathological conclusions were based on the quantitative scores and incidence data in Table 1, Table 2 and Table 3, whereas therapeutic mast-cell staining was interpreted as qualitative descriptive information only.

### 3.5. Sensitivity Analysis

Because several terminal clinical and histopathological endpoints were based on ordinal or semi-quantitative scoring systems, additional Kruskal–Wallis analyses were performed as sensitivity analyses. The corresponding endpoint-specific results are reported with the relevant outcomes. Longitudinal lesion and erythema scores were analyzed using generalized estimating equations rather than separate Kruskal–Wallis tests at individual time points.

### 3.6. Comparative Summary of Treatment-Associated Responses Among Live Probiotics, Paraprobiotics, and the Combined Formulation

To provide an overall view of the measured outcomes, the major findings across behavioral, gross lesion, organ weight, histopathological, mast-cell, and serum biomarker endpoints are summarized in Table 4. The summary distinguishes statistically supported findings from descriptive or variable responses.

## 4. Discussion

The present study used an exploratory pilot experiment to characterize DNCB-induced dermatitis-like inflammation and evaluated the effects of live probiotics, paraprobiotics, and a combined formulation after disease induction. In contrast to preventive probiotic studies, this work focused on an established and relapse-like dermatitis setting characterized by repeated inflammatory challenge and persistent skin lesions. The pilot experiment informed empirical refinement of the therapeutic protocol but was not a formal optimization or validation study because the induction groups were euthanized on different days. The major findings were as follows: (i) repeated DNCB exposure produced dermatitis-like inflammatory features; (ii) the mixed formulation transiently reduced left-ear erythema at Week 2 after multiplicity correction; (iii) paraprobiotic and mixed formulations reduced spleen weight; and (iv) histopathological and serum biomarker responses were variable and did not consistently distinguish intervention groups from placebo.

AD is a chronic inflammatory skin disorder characterized by epidermal barrier dysfunction, pruritus, and type 2 immune activation. Therefore, treatment evaluation in established disease models provides information that is complementary to prevention-based models. A key methodological feature of this study was the use of repeated DNCB challenge combined with occlusive bandaging to produce dermatitis-like inflammation [2,14]. In the therapeutic experiment, the same left ear received both primary induction and secondary challenge, whereas the contralateral right ear remained non-challenged. The pilot and therapeutic protocols were not identical, and direct comparison of the two pilot schedules is limited by their different sacrifice days.

Consistent with dermatitis-like pathology, DNCB exposure induced epidermal hyperplasia, hyperkeratosis, scratching behavior, ear inflammation, splenomegaly, and increased mast-cell numbers in selected tissues of the exploratory pilot experiment [15,16]. Following disease induction, mice underwent a 21-day oral intervention with live probiotics, paraprobiotics, or a combined formulation. Serum IgE did not show statistically significant treatment-associated differences, and IL-4 and IFN-γ remained below the applicable detection limits. Thus, the serum data did not provide evidence of systemic cytokine modulation, and the observed phenotype-level changes should not be interpreted as proof of systemic immune modulation.

During the intervention period, distinct response patterns were observed among the three probiotic-based formulations. However, longitudinal gross-score analysis showed significant group-by-time interactions with limited multiplicity-adjusted post hoc differences. Only the Mix + DNCB group at Week 2 showed a significantly lower left-ear erythema score than the Placebo + DNCB group after Holm correction; no adjusted difference was detected after the secondary challenge. Therefore, the data do not support descriptions of sustained or stable erythema improvement. Although the Mix + DNCB group had the lowest absolute cumulative scratching time among the DNCB-induced groups, it was not significantly different from the Placebo + DNCB group and should not be interpreted as definitive evidence of reduced pruritus-like behavior.

Histopathological group-level interpretation was based on quantitative lesion scores, incidence data, and statistical comparisons in Table 1, Table 2 and Table 3 rather than on representative images. Most intervention-versus-placebo histopathological comparisons were not statistically significant, and dorsal dermal/subcutaneous inflammation was higher in the Paraprobiotic + DNCB group than in the Placebo + DNCB group. In the therapeutic experiment, mast-cell staining was evaluated qualitatively only and did not provide quantitative evidence of treatment efficacy. Spleen enlargement and spleen weight were reduced in the Paraprobiotic + DNCB and Mix + DNCB groups. However, because serum IgE, IL-4, and IFN-γ did not clearly distinguish treatment effects, these spleen-related findings should not be overinterpreted as direct evidence of systemic immunomodulation.

Within the intervention arms, selected endpoint-specific differences were observed, including lower spleen indices in the paraprobiotic and mixed-formulation groups and a lower challenged-left-ear index in the paraprobiotic group. These observations are consistent with the emerging concept that non-viable microbial preparations may retain biological activity [17,18]. Based on previous studies, one possible explanation is that heat-killed bacteria may retain microbial structural components that could interact with host pattern-recognition receptors, such as Toll-like receptors or NOD-like receptors [19,20]. However, this remains a mechanistic hypothesis in the present study because receptor-level activation, downstream signaling analysis, local cytokine profiling, and skin barrier proteins were not examined.

From an application perspective, paraprobiotic preparations may also have practical advantages because their biological consistency is not dependent on bacterial viability. However, formulation stability was not directly evaluated in the present study. Therefore, the potential translational advantages of paraprobiotics should be confirmed in future formulation and stability studies.

The gut–skin axis has been proposed as a conceptual framework linking intestinal microbiota with cutaneous immune regulation [21,22,23]. Previous studies showing immunomodulatory activity of the probiotic strains used in this work provide a rationale for evaluating these strains in inflammatory skin disease [24,25,26,27]. However, because the present study did not assess gut microbiota composition, microbial metabolites, or gut–skin axis-related mechanisms, any involvement of the gut–skin axis remains speculative and should be further investigated in future studies. Mechanistic interpretation should be limited to phenotypic associations observed in this AD-like model.

This study has several limitations. First, although selected serum immunological biomarkers, including IgE, IL-4, and IFN-γ, were measured to explore systemic immune responses, these markers did not show clear treatment-associated differences at the experimental endpoint. Therefore, the current dataset does not provide sufficient evidence to define the systemic cytokine-mediated mechanisms underlying the observed effects. In addition, local skin immune responses, skin barrier-related proteins, gut microbiota composition, microbial metabolites, receptor-level activation, and downstream signaling pathways were not examined. Further studies incorporating tissue-specific cytokine profiling, barrier function analysis, microbiome assessment, and mechanistic validation will be needed to clarify how live, heat-killed, and combined probiotic formulations influence AD-like skin inflammation. Additional limitations include the absence of independent validation of the empirically modified therapeutic induction protocol, the use of male mice only, limited exact reproducibility of the undisclosed proprietary strain ratio, and the lack of formal mast-cell quantification in the therapeutic experiment. Gross lesion scoring was semi-quantitative, although coded photographs were evaluated independently by blinded assessors.

## 5. Conclusions

In conclusion, this study suggests that live probiotics, paraprobiotics, and a combined formulation produced endpoint-dependent responses in a DNCB-induced dermatitis-like model. The mixed formulation transiently reduced left-ear erythema at Week 2, whereas paraprobiotics and the combined formulation showed statistically supported responses in selected organ-related outcomes. However, mechanistic conclusions regarding systemic immunomodulation, gut–skin axis interactions, or pattern-recognition receptor pathways cannot be drawn based on the current dataset. The lack of clear serum biomarker differences despite local gross and histopathological changes highlights the importance of multidimensional evaluation strategies in AD-like animal models. These findings support further investigation of microbial-based interventions for inflammatory skin disease, including paraprobiotic formulations, while emphasizing the need for endpoint-specific interpretation and mechanistic validation.

## Figures and Tables

**Table 1:** Histopathological lesion scores in the right ear across treatment groups.

Organ	Histopathological Finding	Group					
		Healthy Control	Placebo + DNCB	Probiotic + DNCB	Paraprobiotic + DNCB	Mix + DNCB	Eta-Square ^4^
Right ear	Hyperkeratosis/serocellular pustule, epidermis, moderate to severe/high ^1^	0/7 ^2^ 0.0 ± 0.0 ^3^	2/10 0.3 ± 0.7	3/10 0.4 ± 0.7	1/10 0.2 ± 0.6	4/10 0.5 ± 0.7	0.073
	Hyperplasia, epidermis	0/7 0.0 ± 0.0	2/10 0.4 ± 0.8	2/10 0.3 ± 0.7	1/10 0.2 ± 0.6	4/10 0.5 ± 0.7	0.066
	Inflammation, dermis and subcutis	0/7 0.0 ± 0.0	2/10 0.5 ± 1.1	3/10 0.6 ± 1.1	1/10 0.3 ± 0.9	4/10 0.9 ± 1.2	0.089
	Ulceration, necrotizing, dermis Mean score	0/7 0.0 ± 0.0	0/10 0.0 ± 0.0	1/10 0.1 ± 0.3	1/10 0.2 ± 0.6	1/10 0.2 ± 0.6	0.045
	Subtotal of Mean score	0.0 ± 0.0	0.3 ± 0.6	0.4 ± 0.7	0.2 ± 0.7	0.5 ± 0.7	

^1^ Degree of lesions was graded from one to five depending on severity: 1 = minimal (<1%); 2 = slight (1–25%); 3 = moderate (26–50%); 4 = moderate/severe (51–75%); 5 = severe/high (76–100%). ^2^ Incidence: affected mice/total examined mice. ^3^ The final numerical score was calculated by dividing the sum of lesions of affected mice by the total number of examined mice. Data were expressed as mean ± standard deviation (SD). ^4^ η^2^: effect size expressed as eta-squared (η^2^), calculated as the proportion of total variance explained by group differences.

**Table 2:** Histopathological lesion scores in the left ear across treatment groups.

Organ	Histopathological Finding	Group					Eta-Square ^4^
		Healthy Control	Placebo + DNCB	Probiotic + DNCB	Paraprobiotic + DNCB	Mix + DNCB	
Left ear	Hyperkeratosis/serocellular pustule, epidermis, moderate to severe/high ^1^	0/7 ^2^ 0.0 ± 0.0 ^3^	9/10 1.6 ± 1.0 **	9/10 1.4 ± 0.8 **	7/10 0.8 ± 1.0	7/10 1.3 ± 1.2 **	0.261
	Hyperplasia, epidermis Mean score	0/7 0.0 ± 0.0	9/10 1.6 ± 0.7 ***	9/10 1.3 ± 0.7 ***	9/10 1.4 ± 0.8 ***	9/10 1.3 ± 0.8 ***	0.394
	Inflammation, dermis and subcutis	0/7 0.0 ± 0.0	9/10 2.5 ± 1.2 ***	10/10 2.4 ± 1.1 ***	9/10 2.3 ± 1.1 ***	10/10 2.2 ± 1.3 ***	0.448
	Ulceration, necrotizing, dermis	0/7 0.0 ± 0.0	7/10 1.2 ± 0.9 **	7/10 1.1 ± 0.9 *	5/10 0.8 ± 1.0	6/10 1.0 ± 1.1	0.188
	Subtotal of Mean score	0.0 ± 0.0	1.7 ± 0.8 ***	1.6 ± 0.8 ***	1.3 ± 0.9 **	1.5 ± 1.0 ***	

^1^ Degree of lesions was graded from one to five depending on severity: 1 = minimal (<1%); 2 = slight (1–25%); 3 = moderate (26–50%); 4 = moderate/severe (51–75%); 5 = severe/high (76–100%). ^2^ Incidence: affected mice/total examined mice. ^3^ The final numerical score was calculated by dividing the sum of lesions of affected mice by the total number of examined mice. Data were expressed as mean ± standard deviation (SD). * *p* < 0.05, ** *p* < 0.01, and *** *p* < 0.001 compared with the healthy control group. ^4^ η^2^: effect size expressed as eta-squared (η^2^), calculated as the proportion of total variance explained by group differences.

**Table 3:** Histopathological scores of dorsal skin following probiotic intervention in the DNCB-induced dermatitis model.

Organ	Histopathological Finding	Group					Eta-Square ^4^
		Healthy Control	Placebo + DNCB	Probiotic + DNCB	Paraprobiotic + DNCB	Mix + DNCB	
Dorsal skin	Hyperkeratosis/serocellular pustule, epidermis, moderate to severe/high ^1^ Mean score	0/7 ^2^ 0.0 ± 0.0 ^3^	1/10 0.2 ± 0.6	1/10 0.2 ± 0.6	1/9 0.1 ± 0.3	0/10 0.0 ± 0.0	0.050
	Hyperplasia, epidermis Mean score	0/7 0.0 ± 0.0	5/10 1.7 ± 1.3 **	7/10 1.7 ± 1.3 **	4/9 1.1 ± 1.4	6/10 1.4 ± 1.3 *	0.180
	Inflammation, dermis and subcutis Mean score	0/7 0.0 ± 0.0	1/10 0.2 ± 0.6	1/10 0.3 ± 0.9	4/9 1.0 ± 1.2 *^#^	2/10 0.4 ± 0.8	0.131
	Ulceration, necrotizing, dermis Mean score	0/7 0.0 ± 0.0	0/10 0.0 ± 0.0	1/10 0.2 ± 0.6	0/9 0.0 ± 0.0	0/10 0.0 ± 0.0	0.090
	Subtotal of Mean score	0.0 ± 0.0	0.5 ± 0.5	0.6 ± 0.7 *	0.6 ± 0.7 *	0.5 ± 0.5	

^1^ Degree of lesions was graded from one to five depending on severity: 1 = minimal (<1%); 2 = slight (1–25%); 3 = moderate (26–50%); 4 = moderate/severe (51–75%); 5 = severe/high (76–100%). ^2^ Incidence: affected mice/total examined mice. ^3^ The final numerical score was calculated by dividing the sum of lesions of affected mice by the total number of examined mice. Data were expressed as mean ± standard deviation (SD). * *p* < 0.05, ** *p* < 0.01, and *** *p* < 0.001 compared with the healthy control group; # *p* < 0.05 compared with the Placebo + DNCB group. ^4^ η^2^: effect size expressed as eta-squared (η^2^), calculated as the proportion of total variance explained by group differences.

**Table 4:** Comparative summary of treatment-associated responses among live probiotics, paraprobiotics, and the combined formulation in the DNCB-induced dermatitis-like mouse model.

Endpoint	Probiotic + DNCB	Paraprobiotic + DNCB	Mix + DNCB
Scratching behavior	—/variable	—/variable	Trend ↓
Left-ear erythema	Trend ↓	Variable	↓ at Week 2 only
Response after secondary challenge	Trend ↓	Variable	Trend ↓
Left-ear weight	—	↓	Trend ↓
Spleen weight	Trend ↓	↓	↓
Dorsal gross lesion score	Trend ↓	Trend ↓	Trend ↓
Dorsal skin histopathology	—/variable	↑ inflammation	↓
Left-ear histopathology	Trend ↓	↓	Mixed
Pustule/necrosis-related lesions	Trend ↓	↓	Mixed
Therapeutic mast-cell staining	Qualitative only	Qualitative only	Qualitative only
Serum IgE, IL-4, IFN-γ	NS	NS	NS

Abbreviations: ↓ indicates a statistically supported reduction based on the corresponding analysis. “↑ inflammation” indicates a statistically supported increase in dorsal dermal/subcutaneous inflammation relative to the Placebo + DNCB group. “Trend ↓” indicates a numerical or time-dependent decrease that did not clearly distinguish the intervention group from the Placebo + DNCB group. “Variable” indicates inconsistent patterns across time points or tissues. “Qualitative only” indicates representative descriptive observations without formal group-level quantification. NS, not significant; —, no clear treatment-associated reduction.

## Data Availability

The datasets generated and/or analyzed during the current study are available from the corresponding author upon reasonable request.

## Supplementary Materials

The following supporting information can be downloaded at: https://www.mdpi.com/article/10.3390/nu18152419/s1. Figure S1. Representative histopathological features of ear tissues in the exploratory DNCB-induced dermatitis-like model. (A) Representative hematoxylin and eosin (H&E)-stained ear sections from the healthy control, continuous-induction, and intermittent-induction groups. DNCB-treated tissues illustrate dermatitis-like histopathological features, including epidermal hyperplasia, hyperkeratosis, inflammatory-cell infiltration, serocellular pustules, and focal necrosis (arrows). (B) Representative toluidine blue-stained ear sections illustrating mast-cell distribution in the pilot model-development experiment. Formal mast-cell counting for the pilot experiment is reported in Figure 3. Original magnifications (100× and 400×) and scale bars are indicated in the individual image panels. Figure S2. Representative histopathological features of dorsal skin tissues in the exploratory DNCB-induced dermatitis-like model. (A) Representative hematoxylin and eosin (H&E)-stained dorsal skin sections from the healthy control, continuous-induction, and intermittent-induction groups. DNCB-treated tissues illustrate dermatitis-like histopathological features, including epidermal hyperplasia, hyperkeratosis, inflammatory-cell infiltration, serocellular pustules, and focal necrosis (arrows). (B) Representative toluidine blue-stained dorsal skin sections illustrating mast-cell distribution in the pilot model-development experiment. Formal mast-cell counting for the pilot experiment is reported in Figure 3. Original magnifications (100× and 400×) and scale bars are indicated in the individual image panels. Figure S3. Representative progression of dorsal skin lesions and total gross lesion scores in DNCB-induced dermatitis-like mice. (A) Representative dorsal skin appearances of mice at the indicated experimental time points. (B) Total dorsal gross lesion scores from Weeks 0–4. The total score was calculated as the sum of erythema, dryness, edema, and erosion/excoriation scores. Data are presented as mean ± standard deviation. Longitudinal scores from Weeks 1–4 were analyzed using generalized estimating equations with an exchangeable working-correlation structure and robust standard errors, including group, time, and group × time interaction, with mouse identity specified as the repeated subject. Post hoc comparisons between each intervention group and the Placebo + DNCB group at each week were adjusted using the Holm procedure. A significant group × time interaction was detected; however, no intervention-versus-placebo comparison remained significant after Holm correction. Figure S4. Representative H&E staining of dorsal skin in the therapeutic DNCB-induced dermatitis-like mouse experiment. Representative sections illustrate the range of dorsal skin histopathological features observed across groups, including epidermal hyperplasia, hyperkeratosis, serocellular pustules/necrosis, and inflammatory-cell infiltration. The grade shown in an individual representative image should not be interpreted as the grade of the entire treatment group. Group-level interpretation was based on the quantitative lesion scores, incidence data, and statistical comparisons presented in Table 3. Arrows indicate necrotic lesions. Scale bars are shown in the individual figure panels. Figure S5. Representative H&E staining of bilateral ear tissues in the therapeutic DNCB-induced dermatitis-like mouse experiment. Representative sections illustrate histopathological features in the challenged left ear and the non-challenged contralateral right ear. The left ear received primary DNCB induction and secondary challenge, whereas the right ear did not receive local DNCB exposure in the therapeutic experiment. Image-specific histopathological features should not be interpreted as definitive group-level grades. Group-level interpretation was based on the quantitative lesion scores, incidence data, and statistical comparisons presented in Table 1 and Table 2. Arrows indicate necrotic lesions. Original magnification: 400×. Figure S6. Representative toluidine blue staining of dorsal skin in the therapeutic DNCB-induced dermatitis-like mouse experiment. Representative toluidine blue-stained dorsal skin sections are shown to illustrate mast-cell distribution across groups. In the therapeutic experiment, mast-cell staining was evaluated qualitatively, and no formal group-level quantitative mast-cell comparison was performed. Therefore, these images are presented as descriptive observations only and should not be used as evidence of treatment-associated reduction in mast-cell infiltration. Arrows indicate mast cells. Scale bars are shown in the individual figure panels. Figure S7. Representative toluidine blue staining of bilateral ear tissues in the therapeutic DNCB-induced dermatitis-like mouse experiment. Representative toluidine blue-stained sections are shown to illustrate mast-cell distribution in the challenged left ear and the non-challenged contralateral right ear. In the therapeutic experiment, mast-cell staining was evaluated qualitatively, and no formal group-level quantitative mast-cell comparison was performed. These images are therefore presented as descriptive observations only and should not be used as evidence of treatment-associated reduction in mast-cell infiltration. Arrows indicate mast cells. Original magnification: 400×.

## References

[1] Torres T., Ferreira E.O., Gonçalo M., Mendes-Bastos P., Selores M., Filipe P. (2019). Update on atopic dermatitis. Acta Med. Port..

[2] Weidinger S., Novak N. (2016). Atopic dermatitis. Lancet.

[3] Kim J., Kim B.E., Leung D.Y.M. (2019). Pathophysiology of atopic dermatitis: Clinical implications. Allergy Asthma Proc..

[4] Brunner P.M., Guttman-Yassky E., Leung D.Y. (2017). The immunology of atopic dermatitis and its reversibility with broad-spectrum and targeted therapies. J. Allergy Clin. Immunol..

[5] Lan Y.A., Guo J.X., Yao M.H., Liao Z.R., Jing Y.H. (2026). Histamine and pruritus: From molecular mechanisms to clinical therapy. Cell Biochem. Funct..

[6] Puar N., Chovatiya R., Paller A.S. (2021). New treatments in atopic dermatitis. Ann. Allergy Asthma Immunol..

[7] Husein-ElAhmed H., Steinhoff M. (2023). Meta-analysis on preventive and therapeutic effects of probiotic supplementation in infant atopic dermatitis. J. Dtsch. Dermatol. Ges..

[8] Wang F., Wu F., Chen H., Tang B. (2023). The effect of probiotics in the prevention of atopic dermatitis in children: A systematic review and meta-analysis. Transl. Pediatr..

[9] Xue X., Yang X., Shi X., Deng Z. (2023). Efficacy of probiotics in pediatric atopic dermatitis: A systematic review and meta-analysis. Clin. Transl. Allergy.

[10] Chen L., Ni Y., Wu X., Chen G. (2022). Probiotics for the prevention of atopic dermatitis in infants from different geographic regions: A systematic review and meta-analysis. J. Dermatol. Treat..

[11] Li Y., Zhang B., Guo J., Cao Z., Shen M. (2022). The efficacy of probiotics supplementation for the treatment of atopic dermatitis in adults: A systematic review and meta-analysis. J. Dermatol. Treat..

[12] Choi C.Y., Kim Y.H., Oh S., Lee H., Kim J., Park S., Kim J., Chun T. (2017). Anti-inflammatory potential of a heat-killed *Lactobacillus* strain isolated from kimchi on house dust mite-induced atopic dermatitis in NC/Nga mice. J. Appl. Microbiol..

[13] Chen H.-Y., Chen Y.-T., Li K.-Y., Huang H.-W., Lin Y.-C., Chen M.-J. (2022). A heat-killed probiotic mixture regulates immune T cell balance and IgE production in house dust mite extract -induced atopic dermatitis mice. Microorganisms.

[14] Mollanazar N.K., Smith P.K., Yosipovitch G. (2016). Mediators of chronic pruritus in atopic dermatitis: Getting the itch out?. Clin. Rev. Allergy Immunol..

[15] Riedl R., Kuhn A., Hupfer Y., Hebecker B., Peltner L.K., Jordan P.M., Wallert M. (2024). Characterization of different inflammatory skin conditions in a mouse model of DNCB-induced atopic dermatitis. Inflammation.

[16] Huang W.C., Huang C.H., Hu S., Peng H.L., Wu S.J. (2019). Topical spilanthol inhibits MAPK signaling and ameliorates allergic inflammation in DNCB-induced atopic dermatitis in mice. Int. J. Mol. Sci..

[17] Teame T., Wang A., Xie M., Zhang Z., Yang Y., Ding Q., Gao C., Olsen R.E., Ran C., Zhou Z. (2020). Paraprobiotics and postbiotics of probiotic lactobacilli, their positive effects on the host and action mechanisms: A review. Front. Nutr..

[18] Vinderola G., Sanders M.E., Salminen S. (2022). The concept of postbiotics. Foods.

[19] Jastrząb R., Graczyk D., Siedlecki P. (2021). Molecular and cellular mechanisms influenced by postbiotics. Int. J. Mol. Sci..

[20] Fang H., Robin N., Bhatwa A., Marko D.M., Schertzer J.D. (2023). Microbiota and Nod-like receptors balance inflammation and metabolism during obesity and diabetes. Biomed. J..

[21] Fang Z., Li L., Zhang H., Zhao J., Lu W., Chen W. (2021). Gut microbiota, probiotics, and their interactions in prevention and treatment of atopic dermatitis: A review. Front. Immunol..

[22] Wang Y., Wang B., Sun S., Wang Z. (2024). Mapping the relationship between atopic dermatitis and gut microbiota: A bibliometric analysis, 2014–2023. Front. Microbiol..

[23] Xue P., Qin H., Qin D., Liu H., Li J., Jin R., Xiao X. (2023). The efficacy and safety of oral microecological agents as add-on therapy for atopic dermatitis: A systematic review and meta-analysis of randomized clinical trials. Clin. Transl. Allergy.

[24] Shiu W.-C., Chen B.-Y., Ku Y.-W., Chen P.-W. (2024). Exploring the health-promoting potential: Dietary intervention with live or inactivated *Lactobacillus* gasseri HM1 probiotics in obese mice. J. Funct. Foods.

[25] Chen B.-Y., Liu Z.-S., Lin Y.-S., Lin H.C., Chen P.-W. (2025). Oral administration of heat-killed multi-strain probiotics confers durable protection against antibiotic-resistant primary and recurrent urinary tract infections in a murine model. Antibiotics.

[26] Chen Y.-Y., Liu Z.-S., Chen B.-Y., Tam H.-M.-H., Shia W.-Y., Yu H.-H., Chen P.-W. (2025). Effects of heat-killed probiotic strains on biofilm formation, transcription of virulence-associated genes, and prevention of UTIs in mice. Probiotics Antimicrob. Proteins.

[27] Liu Z.-S., Yang Y.-J., Tsai C.-C., Tung Y.-T., Chen P.-W. (2025). Live GABA-producing and heat-killed probiotic consortium improves sleep and modulates inflammation in an anesthesia- and caffeine-induced sleep disturbance model. Food Biosci..

