# Supplementary material for: Comparative Effects of Live and Heat-Killed Lactobacillus Formulations on DNCB-Induced Dermatitis-like Skin Inflammation in Mice
Source: Nutrients. 2026 Jul 24;18(15):2419. doi: 10.3390/nu18152419 (PMC13468662; doi:10.3390/nu18152419)
Supplement: Supplementary file 1 [file nutrients-18-02419-s001.zip › nutrients-4410946-supplementary.pdf]

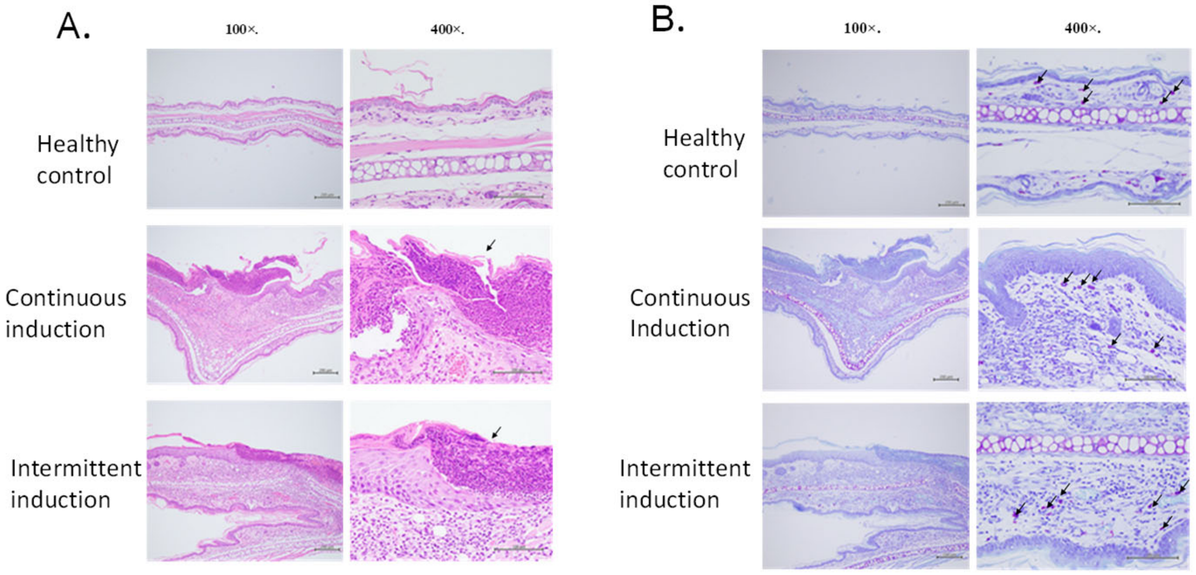

Figure S1. Representative histopathological features of ear tissues in the exploratory DNCB-induced dermatitis-like model.
(A) Representative hematoxylin and eosin (H&E)-stained ear sections from the healthy control, continuous-induction, and intermittent-induction groups. DNCB-treated tissues illustrate dermatitis-like histopathological features, including epidermal hyperplasia, hyperkeratosis, inflammatory-cell infiltration, serocellular pustules, and focal necrosis (arrows).
(B) Representative toluidine blue-stained ear sections illustrating mast-cell distribution in the pilot model-development experiment. Formal mast-cell counting for the pilot experiment is reported in Figure 3. Original magnifications (100× and 400×) and scale bars are indicated in the individual image panels.

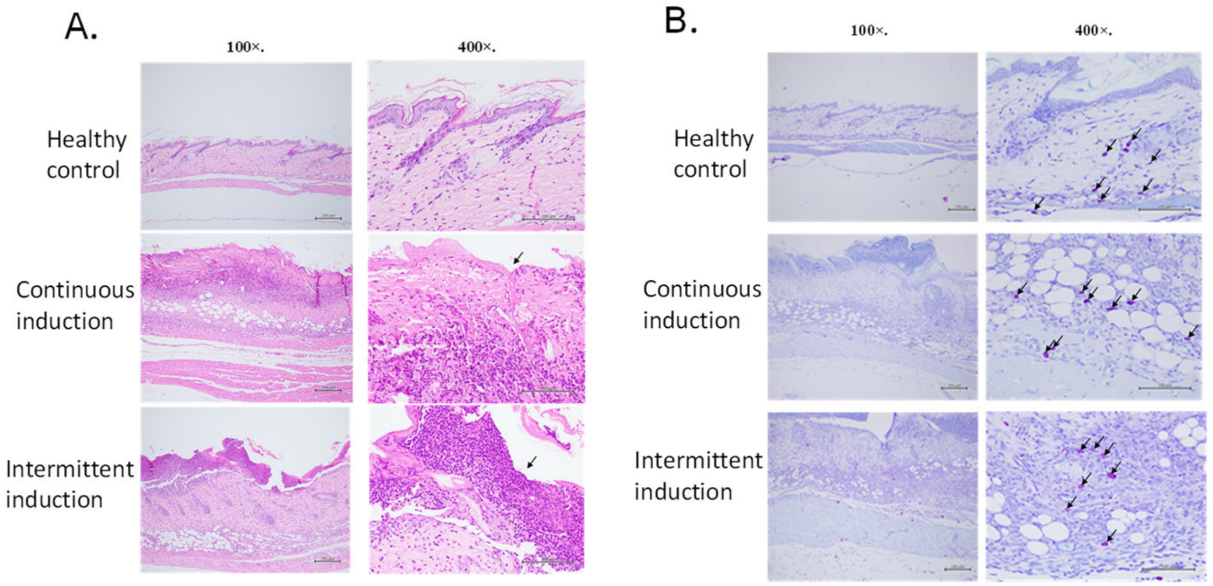

Figure S2. Representative histopathological features of dorsal skin tissues in the exploratory DNCB-induced dermatitis-like model.
(A) Representative hematoxylin and eosin (H&E)-stained dorsal skin sections from the healthy control, continuous-induction, and intermittent-induction groups. DNCB-treated tissues illustrate dermatitis-like histopathological features, including epidermal hyperplasia, hyperkeratosis, inflammatory-cell infiltration, serocellular pustules, and focal necrosis (arrows).
(B) Representative toluidine blue-stained dorsal skin sections illustrating mast-cell distribution in the pilot model-development experiment. Formal mast-cell counting for the pilot experiment is reported in Figure 3. Original magnifications (100× and 400×) and scale bars are indicated in the individual image panels.

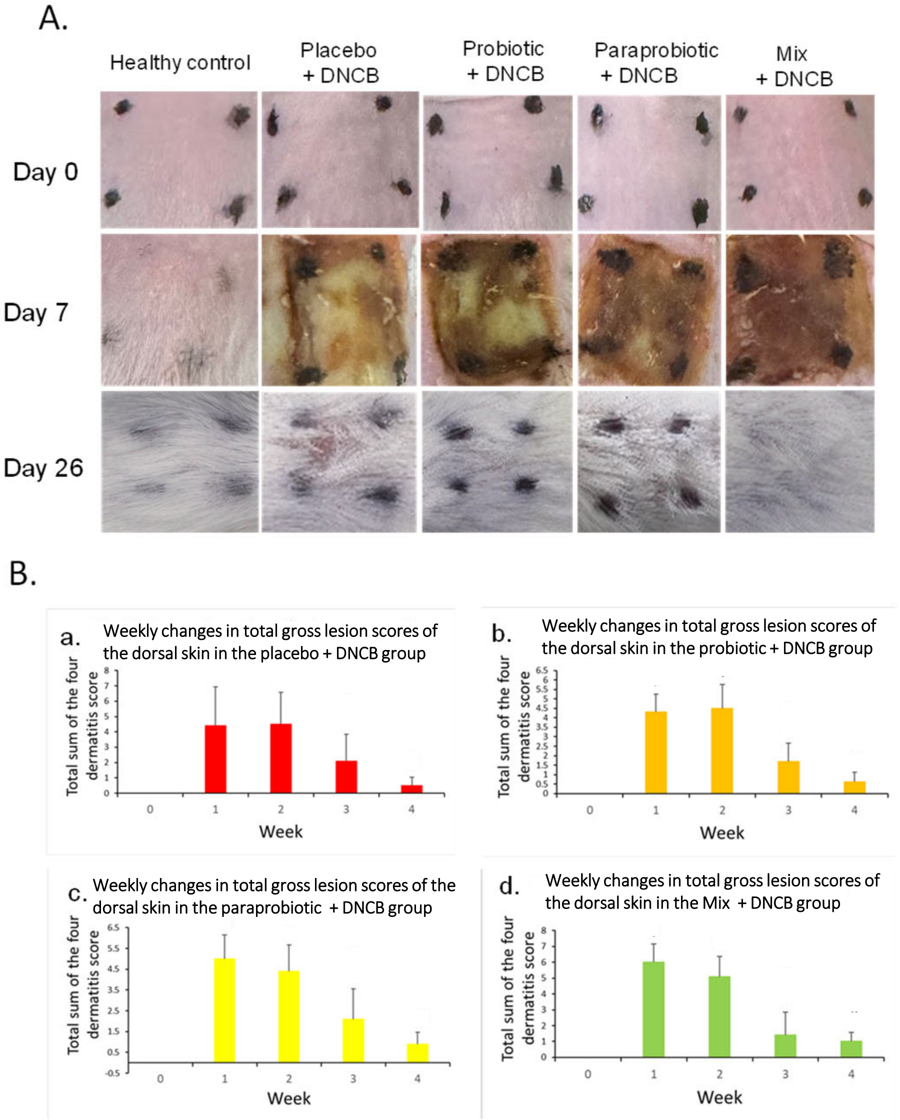

Figure S3. Representative progression of dorsal skin lesions and total gross lesion scores in DNCB-induced dermatitis-like mice.

(A) Representative dorsal skin appearances of mice at the indicated experimental time points.

(B) Total dorsal gross lesion scores from Weeks 0–4. The total score was calculated as the sum of erythema, dryness, edema, and erosion/excoriation scores. Data are presented as mean ± standard deviation. Longitudinal scores from Weeks 1–4 were analyzed using generalized estimating equations with an exchangeable working-correlation structure and robust standard errors, including group, time, and group × time interaction, with mouse identity specified as the repeated subject. Post hoc comparisons between each intervention group and the Placebo + DNCB group at each week were adjusted using the Holm procedure. A significant group × time interaction was detected; however, no intervention-versus-placebo comparison remained significant after Holm correction.

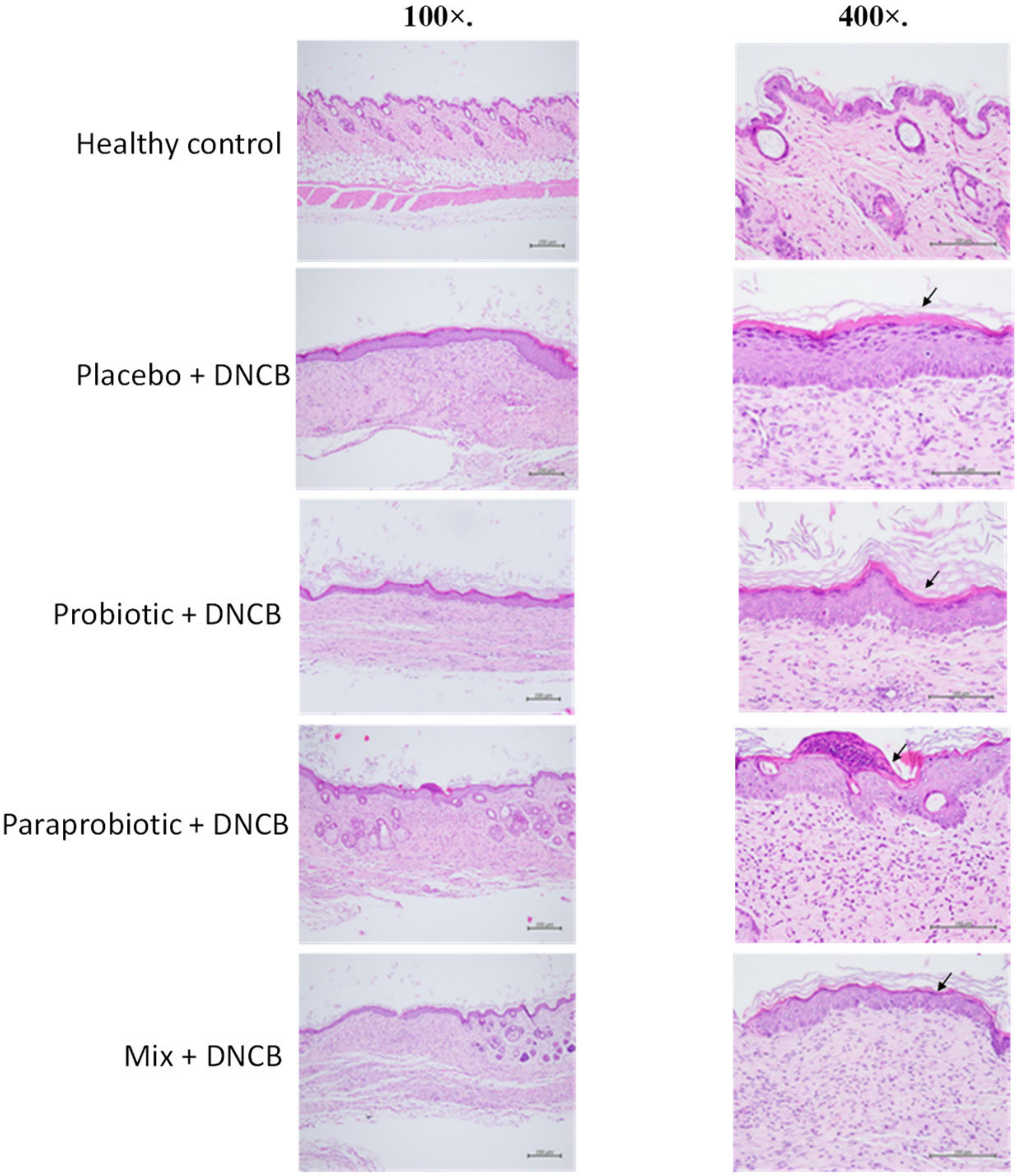

Figure S4. Representative H&E staining of dorsal skin in the therapeutic DNCB-induced dermatitis-like mouse experiment.
Representative sections illustrate the range of dorsal skin histopathological features observed across groups, including epidermal hyperplasia, hyperkeratosis, serocellular pustules/necrosis, and inflammatory-cell infiltration. The grade shown in an individual representative image should not be interpreted as the grade of the entire treatment group. Group-level interpretation was based on the quantitative lesion scores, incidence data, and statistical comparisons presented in Table 3. Arrows indicate necrotic lesions. Scale bars are shown in the individual figure panels.

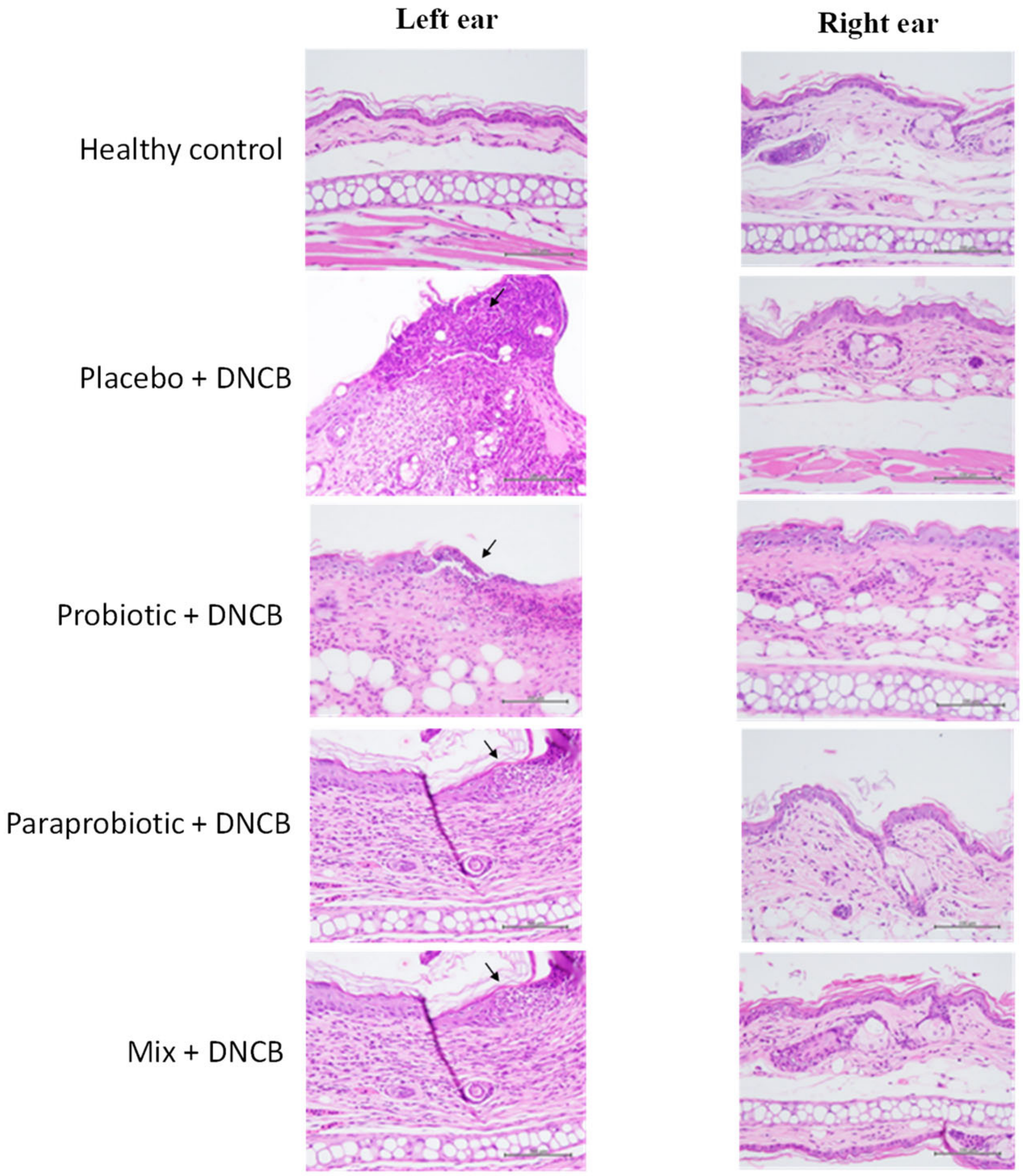

Figure S5. Representative H&E staining of bilateral ear tissues in the therapeutic DNCB-induced dermatitis-like mouse experiment. Representative sections illustrate histopathological features in the challenged left ear and the non-challenged contralateral right ear. The left ear received primary DNCB induction and secondary challenge, whereas the right ear did not receive local DNCB exposure in the therapeutic experiment. Image-specific histopathological features should not be interpreted as definitive group-level grades. Group-level interpretation was based on the quantitative lesion scores, incidence data, and statistical comparisons presented in Tables 1 and 2. Arrows indicate necrotic lesions. Original magnification: 400×.

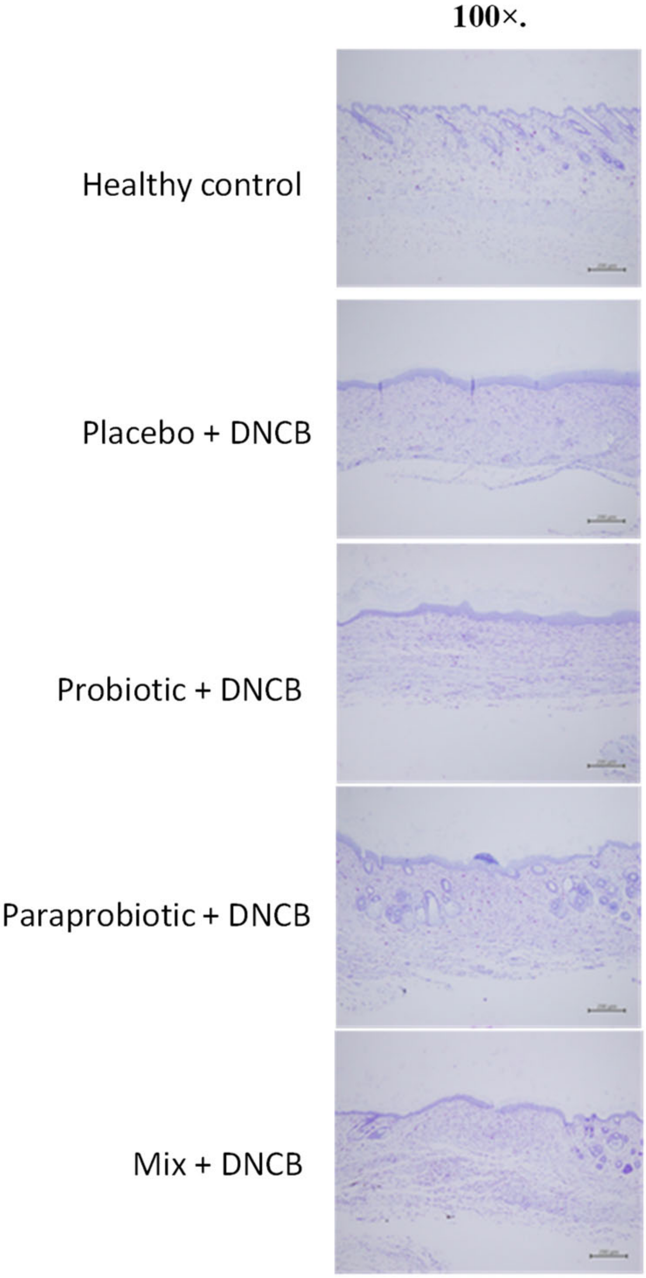

Figure S6. Representative toluidine blue staining of dorsal skin in the therapeutic DNCB-induced dermatitis-like mouse experiment. Representative toluidine blue-stained dorsal skin sections are shown to illustrate mast-cell distribution across groups. In the therapeutic experiment, mast-cell staining was evaluated qualitatively, and no formal group-level quantitative mast-cell comparison was performed. Therefore, these images are presented as descriptive observations only and should not be used as evidence of treatment-associated reduction in mast-cell infiltration. Arrows indicate mast cells. Scale bars are shown in the individual figure panels.

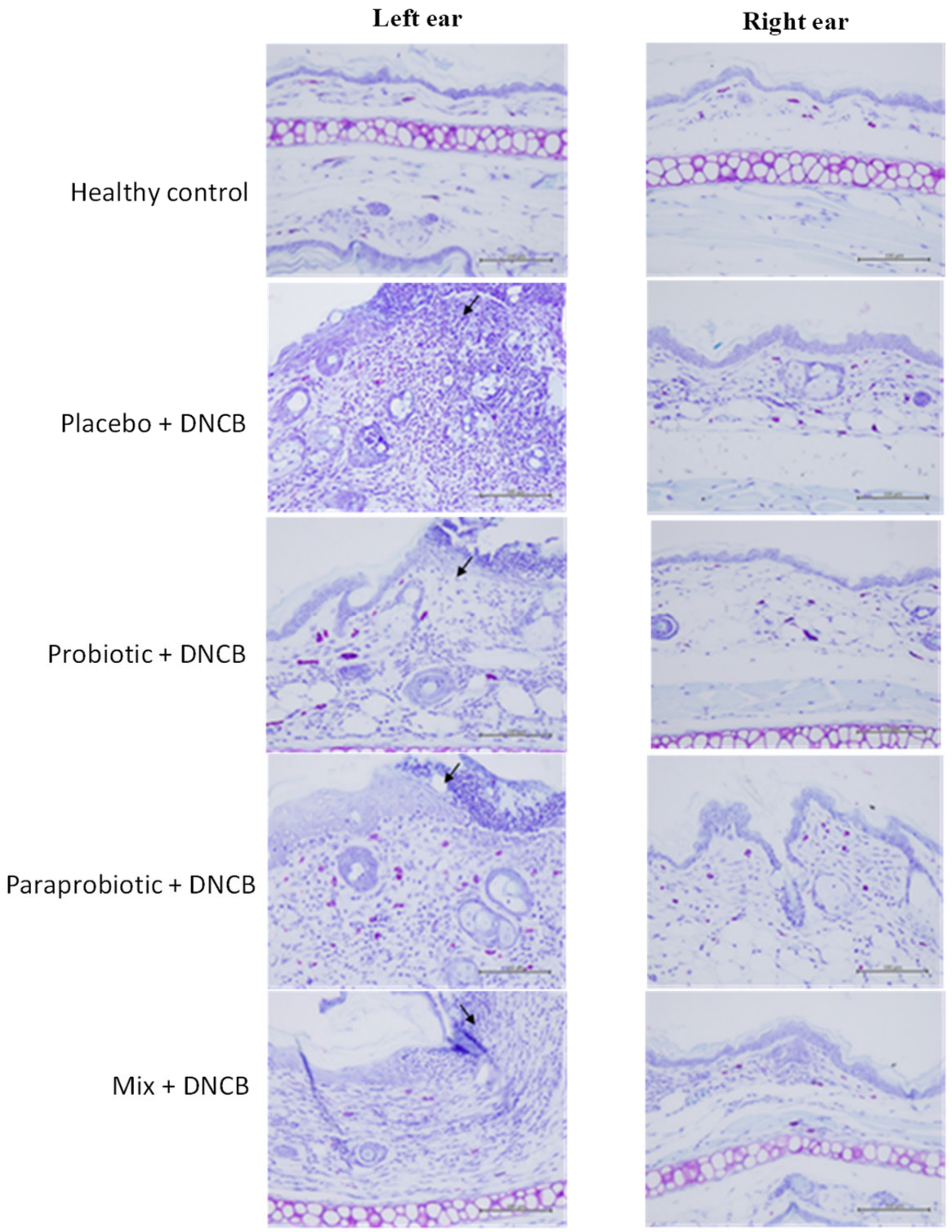

Figure S7. Representative toluidine blue staining of bilateral ear tissues in the therapeutic DNCB-induced dermatitis-like mouse experiment. Representative toluidine blue-stained sections are shown to illustrate mast-cell distribution in the challenged left ear and the non-challenged contralateral right ear. In the therapeutic experiment, mast-cell staining was evaluated qualitatively, and no formal group-level quantitative mast-cell comparison was performed. These images are therefore presented as descriptive observations only and should not be used as evidence of treatment-associated reduction in mast-cell infiltration. Arrows indicate mast cells. Original magnification: 400×.
